# Clinical characteristics of 40 patients with spontaneous pneumomediastinum: a retrospective study and literature review

**DOI:** 10.1186/s13019-026-03885-5

**Published:** 2026-02-03

**Authors:** Hui Tang, Jin Wang, Zhigang Qi, Jiabin Liu, Zhi Liu

**Affiliations:** 1 Emergency Department, Xuanwu Hospital, Capital Medical University, National Clinical Research Center for Geriatric Diseases, No 45 Chang Chun Street, Xicheng District, 100053 Beijing, China; 2 PhaseⅠClinical Trial Center, Beijing Ditan Hospital, Capital Medical University, Beijing, China; 3 Diagnostic Radiology Division, Xuanwu Hospital, Capital Medical University, National Clinical Research Center for Geriatric Diseases, Beijing, China

**Keywords:** High-resolution chest computed tomography, Spontaneous pneumomediastinum, Macklin effect, Clinical characteristics, Literature review

## Abstract

**Objective:**

To investigate the clinical characteristics and imaging features of patients with spontaneous pneumomediastinum (SPM) and conduct a literature review to provide reference strategies for the diagnosis and treatment of the affected population.

**Methods:**

A retrospective analysis was conducted on patients diagnosed with SPM via chest computed tomography (CT) at our Hospital from January 2022 to December 2023. General information, clinical manifestations, and imaging findings were collected and analyzed. Relevant domestic and international literature was reviewed, and a comparative analysis was performed.

**Results:**

A total of 40 patients were included in this study. Acute retrosternal pain was the most common symptom (45.0%), followed by varying degrees of dyspnea, shortness of breath, and chest tightness (25.0%), persistent coughing (17.5%), and discomfort or pain in the neck and throat (5%). The Macklin effect was observed in 38 patients, characterized by varying degrees of gas accumulation around the mediastinum near the pulmonary hilum. After three days of treatment, the symptoms resolved, and follow-up chest CT scans indicated absorption of mediastinal gas. Patients showed improvement and were discharged.

**Conclusion:**

The clinical manifestations of SPM are complex and diverse, making it prone to clinical misdiagnosis or delayed diagnosis, which may hinder timely treatment. Chest CT is instrumental in the accurate diagnosis of mild mediastinal emphysema. A comprehensive evaluation, incorporating patient complaints and clinical symptoms with chest CT findings, is essential for accurate diagnosis.

## Introduction

Pneumomediastinum refers to the presence of free air in the mediastinum, which can occur spontaneously or as a result of secondary causes such as chest trauma, esophageal perforation, or iatrogenic factors. Common symptoms include chest pain, chest tightness, and dyspnea [1]. Pneumomediastinum is categorized into spontaneous and secondary types based on its underlying etiology [2]. Spontaneous pneumomediastinum (SPM) is often associated with intense physical activity, coughing, sudden increases in intra-abdominal pressure, or high‐altitude environments. It is more commonly observed in adolescents and children, particularly those under 18 years old [3]. In contrast, secondary pneumomediastinum is typically caused by trauma, thoracic surgery, pulmonary embolism, or tracheal intubation [4]. In severe cases, pneumomediastinum may compress mediastinal organs and even lead to tension pneumomediastinum, posing a life‐threatening risk [5]. Clinical diagnosis primarily relies on chest X‐ray or computed tomography (CT), with CT providing a clear visualization of gas distribution in the mediastinum [6].

The Macklin effect appears on thoracic CT as linear collections of air contiguous to the bronchovascular sheaths [7]. With the widespread use of pulmonary CT in emergency settings, particularly during the COVID-19 pandemic, the Macklin effect has been increasingly identified in patients presenting with chest tightness and shortness of breath. However, research specifically focusing on pneumomediastinum remains limited. To enhance the understanding of this condition and reduce the likelihood of misdiagnosis or delayed diagnosis, this study retrospectively analyzed the clinical data of patients diagnosed with SPM via CT during emergency evaluations at X Hospital from January 2022 to December 2023. By summarizing the chest CT imaging features and clinical characteristics of pneumomediastinum, this study aims to assist emergency and related department physicians in the early identification and management of the condition.

## Materials and methods

### General information

This retrospective study analyzed the medical records of 40 patients diagnosed with SPM via chest CT at our Hospital from January 2022 to December 2023. Inclusion and exclusion criteria are as follows: (1) Patients meeting the diagnostic criteria for SPM and with complete clinical data were deemed eligible for this study. (2) Cases of secondary pneumomediastinum were excluded. All patients underwent chest CT for further analysis of their clinical symptoms, signs, and CT imaging findings. All chest CT scans were independently reviewed and the diagnosis of spontaneous pneumomediastinum (SPM) was confirmed by two board-certified radiologists with over 10 years of experience in thoracic imaging. The study has been approved by the hospital’s ethics committee.

### Equipment and methods

Chest CT was performed using a GE 64-slice CT scanner (scanning parameters: 120 kV, 300 mA, slice thickness = 5 mm). The scanning range extended from the upper cervical vertebrae to the bilateral adrenal glands to provide a comprehensive and accurate depiction of the location and extent of pneumomediastinum. Before scanning, patients were given respiratory training to minimize motion artifacts and ensure imaging quality.

### Treatment methods

Upon admission, patients received a combination of therapies, including oxygen inhalation, nebulization therapy twice daily using a mixture of 2 mg budesonide suspension, 5 mg terbutaline sulfate solution, and 0.5 mg ipratropium bromide solution to alleviate sputum production, bronchospasms, and airway obstruction, intravenous administration of 30 mg ambroxol hydrochloride once daily to facilitate sputum clearance, and modified traditional Chinese medicine therapy (center-supplementing and qi‐boosting decoction) to support systemic recovery. Therapeutic efficacy was monitored through daily clinical assessment and follow-up imaging.

## Results

### Clinical data

**Fig. 1:**
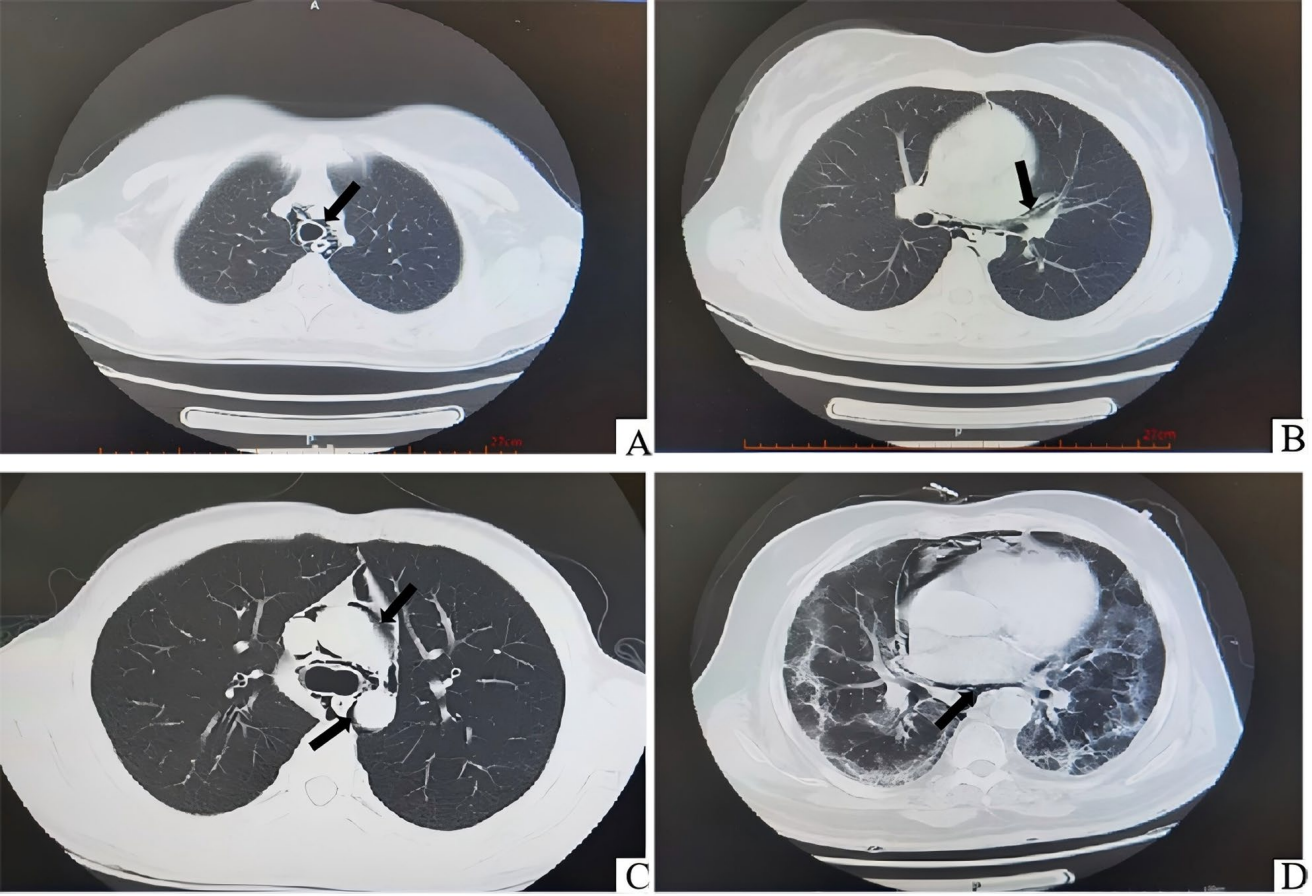
Representative chest CT images of spontaneous pneumomediastinum (SPM) from four different patients, illustrating the Macklin effect. (**A**) Gas surrounding the trachea in the upper mediastinum (indicated by the black arrow) in a patient with SPM triggered by an asthma attack (Patient 12). (**B**) Linear band of air parallel to the tracheobronchial sheath in the middle mediastinum (indicated by the black arrow) in a patient with idiopathic SPM (Patient 5). (**C**) Gas encircling mediastinal vessels and bronchi in the middle mediastinum (indicated by the black arrow) in a patient with SPM following persistent coughing (Patient 28). (**D**) Interstitial lung abnormalities, possibly sequelae of a prior acute viral infection, associated with pneumomediastinum in the lower mediastinum (indicated by the black arrow) in a patient with a history of recent COVID-19 infection (Patient 19)

This study included 40 patients diagnosed with SPM via chest CT. The male-to‐female ratio was 29:11, with an average age of 32.00 years (range: 14–80 years). Among these, 19 cases were idiopathic, 4 were due to trauma, 5 resulted from fever and pulmonary infection, which can lead to increased respiratory effort and forceful coughing, elevating intra-alveolar pressure and potentially causing alveolar rupture with air dissection along the bronchovascular sheaths into the mediastinum [8, 9], 4 were caused by asthma attacks, and 2 were attributed to persistent coughing without fever. Other causes included severe abdominal pain with nausea and vomiting induced by diabetic ketoacidosis (*n* = 2), positive‐pressure ventilation (*n* = 1), emotional agitation during an argument (*n* = 1), long‐distance running (*n* = 1), and encephalitis‐induced seizures (*n* = 1). The Macklin effect was observed in 38 patients (Fig. 1), characterized by linear or tubular air collections tracking along the bronchovascular sheaths, predominantly peribronchial and perivascular, with the extent categorized based on the distribution of gas within the mediastinal compartments [10, 11] (Table 1).

**Table 1:** History or triggers of onset

Etiology/Precipitating Factor	Number of Cases	Percentage (%)
Idiopathic (no obvious cause)	19	47.5
Fever and Pulmonary Infection (including 1 case of COVID-19)	5	12.5
Bronchial Asthma	4	10.0
Trauma	4	10.0
Persistent Cough (without fever)	2	5.0
Diabetic Ketoacidosis	2	5.0
Positive Pressure Ventilation	1	2.5
Physical Exertion (Long-distance running)	1	2.5
Emotional Agitation	1	2.5
Seizures	1	2.5
Total	40	100.0

### Main clinical manifestations

Patients often presented with one or more symptoms. Acute retrosternal pain was the most common symptom, observed in 18 cases (45.0%). Other symptoms included varying degrees of dyspnea, shortness of breath, and chest tightness in 10 cases (25.0%), persistent coughing in 7 cases (17.5%), and neck or throat discomfort in 2 cases (5%). Less common symptoms included abdominal pain, nausea, and vomiting associated with diabetic ketoacidosis (*n* = 2, 5%) and dysphagia (*n* = 1, 2.5%) (Table 2).

**Table 2:** Main clinical symptoms of patients

Main Clinical Symptom	Number of Cases	Percentage of Patients (%)*
Chest Pain	18	45.0
Dyspnea / Shortness of Breath / Chest Tightness	10	25.0
Persistent Cough	7	17.5
Neck or Throat Discomfort/Pain	2	5.0
Abdominal Pain, Nausea, Vomiting (DKA-related)	2	5.0
Difficulty Swallowing (Dysphagia)	1	2.5

* Percentage calculated based on the total number of patients (*n* = 40)

### Outcomes

All 40 patients underwent follow-up chest CT scans after three days of treatment. After three days of treatment, the symptoms resolved, and follow-up chest CT scans indicated absorption of mediastinal gas. Patients showed improvement and were discharged. The average hospital stay was 4.2 days (range: 3–7 days). Symptomatic relief was achieved within 48 h for the majority of patients (37/40, 92.5%). Follow-up CT confirmed complete resolution of pneumomediastinum in 39 patients; one patient with severe COVID-19 pneumonia showed no significant improvement and subsequently died due to complications.

## Discussion

The diagnostic pathway for spontaneous pneumomediastinum often begins with chest radiography, which is readily available in emergency settings. However, the diagnostic sensitivity of conventional chest X-ray for SPM is limited [12]. Overlapping mediastinal and spinal structures can obscure subtle collections of free air, leading to potential false-negative interpretations. In contrast, chest computed tomography provides exceptional soft tissue and air resolution in a cross-sectional format, eliminating superimposition. This makes CT vastly superior for detecting minimal pneumomediastinum, for precisely mapping the distribution of gas, and for visualizing the pathognomonic linear air densities along the bronchovascular bundles known as the Macklin effect, which are frequently not visible on plain films [13]. Therefore, in a patient with persistent symptoms suggestive of SPM but a non-diagnostic chest X-ray, a low threshold for proceeding to chest CT is warranted to establish the diagnosis confidently.

SPM is a benign, self-limiting condition that predominantly occurs in young males and is relatively uncommon, with an incidence ranging from 1 in 8,005 to 1 in 42,000 cases [14–16]. Three mechanisms are known to contribute to the development of SPM: (1) disruption of subcutaneous or mucosal barriers leading to gas entry into the mediastinum; (2) gas produced within the mediastinum due to infection by gas‐forming bacteria or microorganisms; and (3) alveolar rupture [17, 18]. Kaneki et al. [19], in their observation of 33 cases of SPM, reported that 79% (26/33) were associated with intense physical activity or coughing, while 21% (7/33) occurred at rest. In this study, the proportion of SPM cases occurring at rest without clear triggers was as high as 47.5% (19/40). Conversely, persistent coughing (2/40, 5%) and intense physical activity (1/40, 2.5%) were less frequently observed, accounting for only 7.5% (3/40) combined.

The most common clinical symptoms of SPM include chest pain, various degrees of dyspnea (e.g., breathlessness, chest tightness), and neck discomfort or pain. Among these, chest pain and dyspnea are the most frequent, with an incidence of 70.9% [20], which aligns with the findings in this study. Chest pain associated with SPM is often similar to that seen in myocardial infarction or pericarditis, though it is typically positional. For instance, the pain may worsen with physical activity or deep breathing and slightly alleviate when the patient leans forward while seated. In the emergency department, physical examination for SPM can include assessing for Hamman’s sign, a characteristic crunching sound synchronous with the heartbeat, which can help confirm the diagnosis. However, in crowded emergency settings, internists may lack the time or the quiet environment required for detailed auscultation. As a result, no positive records of Hamman’s sign were documented in the physical examinations of the 40 patients in this study.

The Macklin effect in SPM is primarily characterized by radiolucent bands parallel to the left side of the heart and thin radiopaque linear bands directed toward the elevated mediastinal pleura. Additional features include radiolucent linear shadows extending from the mediastinum to the neck and gas surrounding mediastinal structures, such as the aorta, trachea, esophagus, or thymus. Kaneiki et al. [19] classified SPM based on the extent and location of free gas in the mediastinum observed on CT into three grades: Grade A (mild): Gas present in the upper mediastinum; Grade B (moderate): Gas in the middle mediastinum; Grade C (severe): Gas in the lower mediastinum. Chest CT plays a critical role in determining whether mediastinal emphysema is caused by esophageal or tracheal perforation [21, 22]. Despite its generally benign and self-limiting nature, SPM warrants consideration, especially in young male patients presenting with chest pain when ECG and routine chest X‐ray findings are unremarkable. In such cases, chest CT is recommended to confirm the presence of SPM [23–25].

A review of domestic literature reveals that while SPM is occasionally encountered in emergency internal medicine, it often fails to receive sufficient attention from either internal medicine or surgical practitioners. Internal medicine physicians often regard mediastinal emphysema as a surgical condition and refer patients to surgery without further concern. Conversely, surgeons may deem the condition too mild for surgical intervention and advise patients to return home for observation or periodic outpatient follow-up. Given that SPM is typically managed conservatively with favorable outcomes [7], there is limited domestic research on the condition. In the current study, the follow‐up of the patients revealed that only one case resulted in mortality due to organ failure following COVID‐19 and secondary bacterial lung infection, during which the SPM showed no significant improvement or absorption. The remaining cases demonstrated good prognoses, consistent with findings from previous studies [26, 27].

In this study, 38 out of 40 cases exhibited the Macklin effect on imaging, all of which had favorable outcomes. However, the relationship between the Macklin effect and prognosis remains contentious. A study involving 698 COVID-19 cases screened via chest CT identified 33 cases with the Macklin effect, of which 90% required ICU admission and mechanical ventilation [28]. Conversely, another study of 21 patients with COVID‐19 and concurrent mediastinal emphysema reported no correlation between the Macklin effect and mortality [29]. In cases of blunt chest trauma‐induced mediastinal emphysema, the Macklin effect was associated with prolonged ICU stays but not with patient mortality [7]. In this study, one patient with severe pneumonia caused by COVID‐19, who presented with the Macklin effect, ultimately succumbed to the condition, consistent with findings in the literature [30].

Okada et al. [31] proposed five key questions to aid emergency physicians in determining whether further observation or hospitalization is necessary, especially to avoid unnecessary admissions: (1) Is the body temperature > 38 °C? (2) Is oxygen saturation < 96%? (3) Are symptoms progressing? (4) Was vomiting present at onset? (5) Is anxiety evident? If any of the above criteria are met, further observation is recommended. In most cases, hospitalization is not required. Symptoms such as neck swelling and chest pain often resolve spontaneously within a few hours after high-flow oxygen therapy. The use of prophylactic broad‐spectrum antibiotics remains debated. Some studies [32, 33] suggest that antibiotics can help reduce the risk of deep neck infections and mediastinal inflammation. However, other studies [34] argue that prophylactic antibiotics do not provide additional benefits unless there are other suspected causes of mediastinal emphysema, such as Boerhaave’s syndrome (esophageal perforation), esophageal necrosis, or mediastinitis.

This study has several limitations. Due to constraints in research time and cost, the number of cases included was relatively small. Additionally, as a retrospective study, the results may be subject to bias. Future research will aim to include a larger sample size and conduct more in-depth analyses.

## Conclusion

The clinical presentation of SPM is complex and diverse. Traditional DR chest X-rays have limited diagnostic utility due to overlapping images, which fail to clearly depict subtle gas shadows or pulmonary lesions. In contrast, chest CT can clearly display small amounts of gas and its distribution, as well as detect lesions not visible on chest X‐rays. Chest CT is instrumental in the accurate diagnosis of mild mediastinal emphysema. A comprehensive evaluation, incorporating patient complaints and clinical symptoms with chest CT findings, is essential for accurate diagnosis.

## Data Availability

All data generated or analysed during this study are included in this article. Further enquiries can be directed to the corresponding author.
